# Bruises

**Published:** 1872-09

**Authors:** 


### BRUISES,

From blows, strokes, falling timbers, and the like, if slight, may be let alone; if severe, deep, or extensive, the swelling, blackness, and blood are all favorable, because they show that the blood comes outward; while if it went inward, as in the brain, for example, death would ensue.

As the result of a bruise, the blood sometimes comes up to the skin, without coming out, spreads like a sheet, and blackens; this is called

### EFFUSION.

Such wounds affect the muscles, the flesh, make them sore, painful, and weak, according to the severity of the injury.

Sometimes, if there be but little life in the system, the parts die, mortification ensues, and there is a

### SLOUGHING, either to be thrown off by nature, detached from the healthy parts, or to be cut out by the surgeon.

### TREATMENT OF BRUISES.

First, prevent inflammation, by having soft rags, five or six thicknesses; lay them in ice-cold water, and spread over the bruise; either remove every five minutes, to be replaced immediately by another always in readiness, or lay over the wet cloth a piece of oiled silk, extending over its edges about an inch, so as to keep in all the steam; this causes a sweating, an evaporating process, which carries off the extra heat rapidly, and this effectually prevents inflammation. If the frequent cold lotions are used, the extremities of the blood-vessels are congealed, and the bleeding also is arrested; when these two conditions are secured, that is, the prevention of inflammation and the arrest of the bleeding, use warm poultices, to keep up the evaporation and the coolness, and the parts will usually heal, if the bowels are kept acting freely every day, and the diet is cooling, that is, mainly of lean meat in small quantities, once a day, with coarse bread and fruits and lemons and melons.

### COLD WATER STREAM.

One of the very best remedies for bruises as well as sprains, is to have a stream of cold water fall on the part until it almost aches with cold, then desist, and renew every third hour until the inflammation subsides; a pitcher or tea-kettle, or old coffee-pot can be used, but this requires the time of another person, which can be obviated by having a barrel of water higher than the head, and attached to it an India rubber tube, which can be stopped with a cock; or if there are water pipes in the house, have a tube large enough to go over the end of the faucet. Bruises are cured and the blood and other parts are absorbed by the application of a bandage, but this requires skill; hence it would be better to use the cold water in one of the ways named; or employ washes or bathings of

### TINCTURE OF ARNICA.

Apply it every hour or two, or keep a rag or lint saturated, kept wet by it, on the part, until the symptoms have abated, and there is a feeling of quiet and comfort.

The concentrated tincture of *rhus ton* may be employed to advantage when joints, tendons, and synovial membranes are injured; but in all cases keep the bowels freely acting every day; this is indispensable, always.

Very painful wounds and bruises are often promptly relieved thus: Take a shovel of burning coals,—of wood is better; sprinkle common brown sugar on the coals, and hold the wounded part in the smoke; in case of splinters or rusty nails piercing the flesh and causing pain, fever, and irritation, the discomfort is sometimes removed in fifteen minutes. Other

WOUNDS AND BRUISES

may be treated successfully in the same way.

Another method of allaying inflammation and modifying the ill effects of metals piercing the flesh and thus preventing

LOCK JAW, mortification, and the necessity of amputation, is, — Unravel a piece of flannel or woolen stocking, or take common woolen yarn, or much worn woolen fabrics, saturate them with sweet oil, hog's lard, or melted butter; put them in a kettle, set them on fire so they shall smoke, without blazing; hold the wound over the smoke, and cover the wounded part with a blanket so as to condense the smoke about the wound, but all done in such a way as not to smoke the sufferer or strangle him to death.

Another method, successfully adopted for many years, consisted of holding the wounded or bruised part over the smoke made of old shoes, or any bits of leather made to burn without blazing; these methods prevent the mortification of the living part, just as the old-fashioned smoke-houses of half a century ago prevented the hams, and sides, or middlings of pork from decaying. In all these cases we have the one thing, the condensed smoke, as the curative agent, known in later years as

CREOSOTE, which is really the essence of smoke or soot. Common tar is made, by setting some kinds of wood on fire, covering it over with earth, so as to prevent it from burning, leaving the more solid wood in the shape of charcoal, while the other part, the tar, flows out, and is gathered and preserved for various uses. When this tar is distilled, it yields creosote.

CARBOLIC ACID

is the tar of coal oil, which is found in that portion of coal tar which distils over, under a heat of three or four hundred degrees; it is a colorless liquid, of an oily look, of a burning taste, and has something of the smell of soot; hence creosote and carbolic acid are of the same nature in some of their qualities, and in their effects on wounds resemble those of the smoke arising from burning leather, rags, etc. But in many places liable to accidents and burns, as on steamboats and railways, and in the interior of the country, where neither of them could be possibly obtained, some old woolen rags, or old shoes, could be got together, and the smoke of them concentrated on wounds, bruises, cuts, crushes, and burns, and be of inestimable value, all having the one effect of

### COAGULATION.

By coagulating the blood it arrests bleeding; it also constringes the extreme ends of the smallest blood-vessels, and thus also arrests the flow of the vital liquid, and heals every variety of wounds and hurts.

Carbolic acid prevents putrefaction; it averts mortification, as well as prevents it; in addition, it takes away all ill odors, and keeps the parts in a cleanly, healthy condition.

One part of carbolic acid, with forty parts of hot water, shaken well, and then strained or filtered, is an excellent wash for all sores, ulcers, bruises, and the like.

A horse was about dying of a festered wound; some old shoes were cut up in a hog trough, and the pieces set on fire under the swollen wound of the horse; in a few hours the swelling began to subside, and to discharge yellow matter, and the horse was saved. Another horse had been gored by a bull, in the abdomen; nothing seemed to be of any avail; the smoke of leather was advised, and the horse got well.

A man's foot was cut with an axe, and while bleeding badly, a lady seized hold of it, held it over the smoke of burning tag-locks, or scraps of leather; in a few moments the bleeding stopped; the wound never mattered, nor was there any pain, and it got well rapidly. The smoke, which contains creosote or carbolic acid, coagulates the albumen, and thus prevents putrefaction; it coagulates the whole blood, and thus arrests bleeding; hence smoke has a valuable healing power when applied to ulcers, sores, and man's skin diseases; but as carbolic acid contains the curative element in the smoke, or rather is itself the curative principle, every family would do well to have it always on hand. To further impress this on the mind of the reader, it is proper to state that a patent has been taken out in Paris for a new agent to stop bleeding, etc., in wounds. The French government has for many years been far in advance of all civilized nations in purchasing valuable secrets from persons who have discovered them, and then making them public to all the world. This new agent is made thus: Take common

PETROLEUM, and stir into it, cold, one sixth of its weight of caustic soda; let it stand twelve hours; it will then be found to have separated into two layers; the lower one is

PHENATE OF SODA.

Run it off, and keep in a glass bottle for use; if a cut, dip several folds of linen or muslin into it, and lay it on the cut; press it on the wound, and then with a rag apply more phenate on the compress; it causes no pain or irritation; then apply a second compress; wash it also with a rag dipped in the phenate; keep on applying a new compress, saturated with the phenate, until the bleeding stops; these compresses are applied, one over the other, until the blood coagulates.

If a wound is made by a bullet, or sharp or other instrument, inject the phenate into it several times, then saturate lint with the phenate, and fill the place with it. The operation of this valuable remedy is

GOOD.

It coagulates the blood. It renders the edges of the wound insensible. It causes the sides of the wound to contract by its constringent power. It contracts also the minute blood-vessels, causing them to send forward what blood they have, thus removing the congestion, and by diminishing their calibres, on the same principle, prevents too much blood from coming into them and through them, to cause inflammation.

### ALOES AND ALCOHOL.

Take one part of Socotrine aloes and two parts of alcohol, dip into this a soft rag, or lint, and lay it on any sore, such as bed-sores, ulceration from burns, or other causes; it often heals without causing any scars. This preparation should be always kept in the house. Aloes is a common drug, a vegetable product, and is used largely in purgative preparations, so that there is no danger whatever in its employment externally.

